# Factors Influencing Saccadic Reaction Time: Effect of Task Modality, Stimulus Saliency, Spatial Congruency of Stimuli, and Pupil Size

**DOI:** 10.3389/fnhum.2020.571893

**Published:** 2020-11-26

**Authors:** Shimpei Yamagishi, Shigeto Furukawa

**Affiliations:** Human Information Science Laboratory, NTT Communication Science Laboratories, Nippon Telegraph and Telephone Corporation, Atsugi, Japan

**Keywords:** saccadic eye movement, spatial attention, pupil size, multisensory integration, orienting behavior, superior colliculus

## Abstract

It is often assumed that the reaction time of a saccade toward visual and/or auditory stimuli reflects the sensitivities of our oculomotor-orienting system to stimulus saliency. Endogenous factors, as well as stimulus-related factors, would also affect the saccadic reaction time (SRT). However, it was not clear how these factors interact and to what extent visual and auditory-targeting saccades are accounted for by common mechanisms. The present study examined the effect of, and the interaction between, stimulus saliency and audiovisual spatial congruency on the SRT for visual- and for auditory-target conditions. We also analyzed pre-target pupil size to examine the relationship between saccade preparation and pupil size. Pupil size is considered to reflect arousal states coupling with locus-coeruleus (LC) activity during a cognitive task. The main findings were that (1) the pattern of the examined effects on the SRT varied between visual- and auditory-auditory target conditions, (2) the effect of stimulus saliency was significant for the visual-target condition, but not significant for the auditory-target condition, (3) Pupil velocity, not absolute pupil size, was sensitive to task set (i.e., visual-targeting saccade vs. auditory-targeting saccade), and (4) there was a significant correlation between the pre-saccade absolute pupil size and the SRTs for the visual-target condition but not for the auditory-target condition. The discrepancy between target modalities for the effect of pupil velocity and between the absolute pupil size and pupil velocity for the correlation with SRT may imply that the pupil effect for the visual-target condition was caused by a modality-specific link between pupil size modulation and the SC rather than by the LC-NE (locus coeruleus-norepinephrine) system. These results support the idea that different threshold mechanisms in the SC may be involved in the initiation of saccades toward visual and auditory targets.

## Introduction

Humans and animals must be able to direct their attention toward objects of interest. Rapid eye movements toward the stimulus, namely saccades, have been studied in relation to how our attentional system is affected by changes in environmental stimuli. In order to optimize orienting behaviors such as making a saccade toward a multisensory object, the sensory information coming from different modalities has to be integrated in the brain. The orienting responses reflect not only external factors, or stimulus-related factors, such as the intensity of sensory stimuli (saliency), but also internal factors such as task modality, arousal state, and neural baseline activity. We can make a saccade not only to visual stimuli but also to auditory stimuli (Zambarbieri et al., 1982; Frens and Van Opstal, 1995; Corneil et al., 2002; Zambarbieri, 2002; Gabriel et al., 2010). It is known that visually driven saccades (visual saccades) and auditory-driven saccades (auditory saccades) have different properties. For example, auditory saccades show lower peak velocity and longer duration than visual ones (Zambarbieri et al., 1982; LaCroix et al., 1990). Previous studies have shown that stimulus saliency affects the saccadic reaction time (SRT) for the visual target (Bell et al., 2006; Marino et al., 2015). However, the effect of stimulus saliency remains controversial for the auditory target: Corneil et al. (2002) reported that SRT decreased as the signal-to-noise ratio of the auditory target increased, but Gabriel et al. (2010) reported no effect of sound intensity on SRT. SRT also reflects the position of the target: the reaction time of the auditory-driven saccade is shorter for a sound originating from an eccentric position than from a position close to the center, but this eccentricity effect was not confirmed for visual saccades (Frens and Van Opstal, 1995; Gabriel et al., 2010). The discrepancy in the stimulus intensity and eccentricity effect between auditory and visual saccades implies that modality-specific mechanisms play dominant roles in determining the contributions of stimulus-related factors to saccadic behaviors.

This inference is also consistent with existing neurophysiological data. The deep layer of the superior colliculus (dSC) in the midbrain receives visual, auditory, and somatosensory inputs and is considered to play a central role in initiating orienting responses such as saccades, head movements, and pinna movements (Sparks, 1986). Previous studies showed the different properties of dSC activity between unimodal auditorily and visual saccades. Visual cues elicit a robust response in the dSC, which is followed by increasing baseline pre-target activity, whereas auditory cues evoke a weaker but earlier onset response and less of an increase in pre-target activity compared with the visual response (Bell et al., 2004). A conceptual model based on the properties of SC activity including such modality specificity can explain the difference in saccade behavior between visual and auditory domains (Corneil et al., 2002).

Recent studies have shown that pupil size or the velocity of pupil-size variation before saccade execution correlates with SRT for visual saccades (Jainta et al., 2011; Wang et al., 2015; 2017), but the correlation is weak or not significant for auditory saccades (Wang et al., 2017). The pupil size tracks locus coeruleus (LC) activities, which are known to reflect the arousal state (Aston-Jones and Cohen, 2005; Gilzenrat et al., 2010). Recent neurophysiological studies have shown direct evidence of a correlation between LC activities and pupil size in monkeys (Joshi et al., 2016) and mice (Breton-Provencher and Sur, 2019). In humans, fMRI studies have shown a relationship between LC BOLD activity and pupil size (Murphy et al., 2014; de Gee et al., 2017). Thus, the study by Wang et al. (2017), which showed a correlation between changes in pupil size and SRT for only visual saccades implies that an internal factor, the effect of the arousal level, may also differ between visual and auditory saccades. It should be noted, however, recent accumulating evidence have suggested that pupil size also correlates with cholinergic (ACh) (Reimer et al., 2016), possibly serotonergic (Cazettes et al., 2020), and SC activities (Wang et al., 2012, 2014; 2015), and thus the neural mechanism underlying the correlation between pupil size and SRT is still unclear. Nevertheless, the preparatory activities related to the pupil size change may differ between sensory modalities.

The aim of this study is to explore the effects of and the interaction between the external stimulus factors and the internal factors on the saccade toward audiovisual objects. We conducted an experiment in a situation where visual and auditory stimuli existed at the same time. We examined the factors influencing the saccade behavior, such as task modality, stimulus saliency, spatial congruency of the audiovisual stimuli, and pupil size. We conducted studies under separate visual- and auditory-target conditions in order to investigate the effect of task modality. As for the visual (auditory) target conditions, the participants were required to make a saccade toward a visual or auditory stimulus. We adopted the so-called “gap paradigm,” in which the fixation point is removed shortly before the appearance of the peripheral target. The SRT in this condition is considerably shorter than in a condition without a gap (Saslow, 1967; Dorris and Munoz, 1995). The offset of the central fixation point reduces the activity of fixation neurons in the SC, which thus releases the visual attention, and the oculomotor system then responds more quickly to new stimuli (Everling et al., 1998). The gap paradigm is suitable for our purpose, which is to compare visual- and auditory-targeting saccades: the gap effect has also been reported in the auditory modality, but the effect is smaller than in the visual condition (Shafiq et al., 1998). Since we wanted to test saccade behavior (e.g., SRT) in a comparable range of target positions between the visual and auditory domains, we selected a target position of 10 degrees, where the eccentricity effect may not be dominant for auditory cues (Gabriel et al., 2010).

## Materials and Methods

### Participants

Fourteen adults participated in the experiments. Their ages ranged from 20 to 49 years (mean = 34.6). None of the participants were diagnosed as having any neurological problems. The experimental protocols were approved by the Research Ethics Committee of Nippon Telegraph and Telephone (NTT) Communication Science Laboratories. All listeners gave written informed consent prior to the experiment.

### Apparatus

**FIGURE 1:**
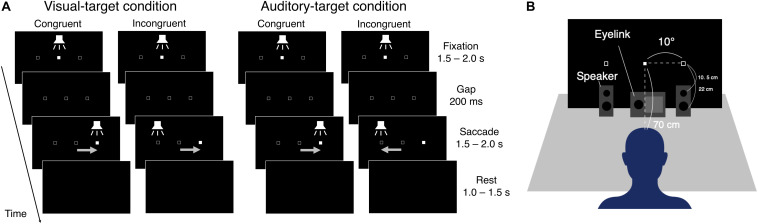
**(A)** Schematic representation of the saccade tasks. Visual- and auditory-target conditions were conducted in separate blocks. First, the visual and auditory stimuli were presented at the center position (Fixation period). The visual stimulus was a filled square (1° on each side). The auditory stimulus was white noise. The visual stimulus was changed to an empty square, and the auditory stimulus disappeared for a period of 200 ms (Gap period), followed by the appearance of the visual or auditory stimuli at one of the peripheral positions (Saccade period). For the saccade period, congruent or incongruent trials occurred randomly. After the Saccade period, all stimuli disappeared (Rest period). **(B)** Experiment setup.

The eye data were recorded with an Eyelink system (ER Research, Toronto, Canada) at a sampling rate of 1000 Hz. Visual stimuli were generated with Matlab (R2016b) and were presented on a monitor with a resolution of 1920 × 1080 pixels and a refresh rate of 60 Hz. Participants sat on a chair and put their heads on a chin rest. The distance between the chin rest and the center of the display was around 70 cm. Auditory stimuli were synthesized with Matlab at a sampling frequency of 44.1 kHz and were presented through a digital-to-analog converter and two loudspeakers (Fostex, PM0.3, Tokyo, Japan). The loudspeakers were placed in front of the display. The loudspeaker has a woofer with a diameter of 7.5 cm and a tweeter with a diameter of 1.9 cm. In the experiment, the horizontal positions of the center of cones were ±10° from the center of the display (the same positions as the visual cue positions, see the next section). The vertical positions of the woofer and the tweeter, respectively, were 5.5 cm and 17 cm above the desk, and 22 cm and 10.5 cm below the visual cue position. The depth positions of the centers of the cones were 13 cm in front of the display (the thickness of the loudspeaker was 13 cm) (Figure 1B).

### Stimuli and Procedure

The present experiment explored the effects of three factors on SRT, namely target saliency (the intensity of the visual or auditory target), target-versus-non-target congruency (whether stimuli in the two modalities point in the same or opposite directions in space), and non-target saliency (intensity of visual or auditory stimuli that was not the targets of the task). It should be noted that visual and auditory stimuli were always presented simultaneously. When the visual cue, for example, was specified as the target for the saccade, the auditory cue was regarded as the non-target stimulus. The participants performed two separate blocks of saccade tasks that differed in the target modality (i.e., the modality to which the task is relevant). The tasks in visual-relevant and auditory-relevant blocks are referred to as *visual-target* and *auditory-target task*s, respectively. We conducted the experiment with a block design because we wanted to compare the effect of task set (“visual mode” vs. “auditory mode”) in terms of saccade behavior and pupil size change. Within each block, the side on which the visual or auditory stimulus was presented (left or right) was randomly and independently varied across trials. Therefore, visual and auditory stimuli were on the same side in half of the trials (“congruent” trials) and on different sides in the other half (“incongruent”). Thus, for example, in an incongruent trial when the visual stimulus was on the left and the auditory stimulus was on the right, a leftward saccade had to be made in the visual-relevant block and a rightward one in the auditory-relevant block. The intensity of the visual or the auditory stimulus (as a factor that would control the stimulus salience) was also varied randomly and independently across trials between two levels for each modality. Thus, there were weak and intense relevant stimuli and weak and intense irrelevant stimuli.

Figure 1A shows the schematic procedure of one trial in the experiment. The background color of the display was black (∼0 cd/m^2^) throughout the experiment. In both tasks, one trial consisted of four periods (Fixation, Gap, Saccade, and Rest). The visual and auditory targets were filled squares (1° each side) and white noise, respectively. Open squares were presented at the to-be-presented positions of the target throughout the experiment, except for the Rest period. In the Fixation period, the participant was instructed to fixate on the center of the display, which was indicated by the visual and auditory stimuli for 1.5–2.0 s. In the Gap period, the central stimuli disappeared for a period of 200 ms. In the Saccade period, the target appeared at the left or right sides of the display (10 degrees from the center of the display in viewing angle). In the Rest period, all visual and auditory stimuli disappeared until the next trial started.

The luminance levels of the weak and intense visual stimuli were 2.58 or 93.58 cd/m^2^, respectively. The A-weighted sound pressure levels of the weak and intense auditory stimuli were 40 and 60 dB, respectively. The visual and the auditory stimuli in the Fixation period were always the same as the weaker targets. The intensity of the auditory target (white noise) was measured with a sound level meter (B & K, Type 2250) equipped with a free-field microphone (B & K, Type 4189) placed at the chin rest. The auditory cue was presented from two speakers placed at the same horizontal positions as the left and right visual target positions. Centralized sound was presented with this stereophonic system by the left and right speakers simultaneously emitting sounds of the same intensity. The experiment setup is schematically shown in Figure 1B.

Participants took part in eight sessions, four in the visual-target condition and four in the auditory-target condition. In each session, 48 trials were conducted. In this experimental design, the number of combinations of factors was eight: 2 (saliency of the target) × 2 (congruency) × 2 (saliency of the non-target stimulus). Thus, we obtained 24 repetitions (4 × 48/8) of data for each combination and each participant. The order of visual- and auditory-target conditions was counterbalanced.

### Detection of Saccades

The onset of a saccade was defined as the time point when the velocity exceeded a certain threshold. The threshold was six times the standard deviation of velocity in one session. We only analyzed the time after the onset of target presentation. The SRT was defined as the interval between the onset of target presentation and the onset of the saccades. The following trials were regarded as error trials and thus were excluded from further analysis: (1) when the saccade direction was opposite to the target direction (e.g., a saccade toward the visual stimulus which was incongruent with auditory stimuli in the auditory-target condition), (2) when the saccade amplitude was smaller than 7 degrees or larger than 13 degrees, (3) when no saccades were detected, and (4) when the SRT was shorter than 70 ms or longer than 1 s. In total, 28.8% of all trials were excluded from the analysis. On average, erroneous saccades (saccade toward the direction opposite to the target) was 10.5% of all trials.

### Analysis of Pupil Data

The Eyelink system outputs the pupil size in arbitrary units that depend on the distance between the participant’s eyes and the camera, etc.; thus, its values should not be directly compared across participants or blocks of trials. This was not a problem for the purpose of the present study, since the present analyses were based on the relative measure derived on a trial-by-trial basis (described in more detail in the “Results”). The pupil data during blinks or unsuccessful recordings were treated as missing data and excluded from the analysis. We also excluded data that were 100 ms before and after the offset of blinks. To examine the effect of saccade preparation on pupil size, we computed the absolute pupil size and the velocity of pupil size (first-order derivative) before target presentation (−0.7 to 0 s from the onset of the saccade target). Pupil derivative was derived by subtracting neighboring samples of pupil size values for each data point. Before calculating derivative, pupil size values were averaged with a 50-ms moving window. We calculated the correlation of SRTs with the pre-saccade pupil size and derivative (average within −0.7 to 0 s from target onset). Since the pupil size in a video image can apparently be distorted depending on the relationship between the camera and gaze directions (Wyatt, 2010; Choe et al., 2016; Hayes and Petrov, 2016), trials with a viewing angle exceeding 5 degrees during this time range were excluded from the analysis.

### Statistical Analysis

We conducted three-way repeated measures ANOVAs separately for the visual- and auditory-target conditions. The three factors were *Sal-target* (saliency of the target stimulus), *Sal-nontarget* (saliency of the non-target stimulus), and *Cong* (spatial congruency of the visual and auditory stimuli). It should be noted that it would not be appropriate to conduct a single three-way ANOVA with target modality as the fourth factor, because the other factors in the visual- and auditory-target conditions should not be treated equally. That is, the salience and spatial position of the stimuli were not directly comparable between the visual and auditory modalities. Statistical comparisons, including the ANOVAs, were conducted on log-transformed SRTs because reaction time data are not normally distributed with a long-tail toward larger values (Rouder et al., 2005). The results of the ANOVAs are summarized in Tables 1, 2 for the visual- and auditory-target conditions, respectively. The sections in Results examine the effects of individual factors and their interactions in accordance with the ANOVA results.

**TABLE 1:** ANOVA results on SRTs for visual task.

***Effect***	***F***	***p***	**$\eta_{\text{p}}^{2}$**
Sal-target	**160.00**	**<0.0001**	**0.92**
Cong	**23.13**	**0.0003**	**0.64**
Sal-nontarget	0.11	0.74	0.0087
Sal-target × Cong	**6.56**	**0.024**	**0.34**
Sal-target × Sal-nontarget	0.12	0.74	0.0088
Cong × Sal-nontarget	0.019	0.89	0.014
Sal-target × Cong × Sal-nontarget	2.05	0.18	0.14

*The bold values mean statistical significance (p < 0.05).*

**TABLE 2:** ANOVA results on error rates for visual task.

***Effect***	***F***	***p***	**$\eta_{\text{p}}^{2}$**
Sal-target	**8.14**	**0.014**	**0.39**
Cong	**15.51**	**0.0017**	**0.54**
Sal-nontarget	4.60	0.052	0.26
Sal-target × Cong	**13.86**	**0.0026**	**0.52**
Sal-target × Sal-nontarget	2.02	0.18	0.13
Cong × Sal-nontarget	1.72	0.21	0.12
Sal-target × Cong × Sal-nontarget	0.06	0.81	0.0045

*The bold values mean statistical significance (p < 0.05).*

It was anticipated that the effects of strong and weak non-targets (i.e., *Sal-nontarget*) on SRT would be opposite when the non-target was congruent and incongruent. A stronger non-target would enhance the response (hence, shorten SRT) in the congruent condition but would distract it in the incongruent one. When we evaluated the effect of *Sal-nontarget*, we were interested in the size of this enhancement/distraction rather than non-target saliency *per se*. Therefore, the order of SRTs for the strong and weak non-target was reversed in the incongruent condition when the data underwent the ANOVA. That is, a significant main effect of *Sal-nontarget* would indicate that a stronger non-target enhances and distracts the response in the congruent and incongruent conditions, respectively, more than a weaker one does.

## Results

### Visual-Target Condition

**FIGURE 2:**
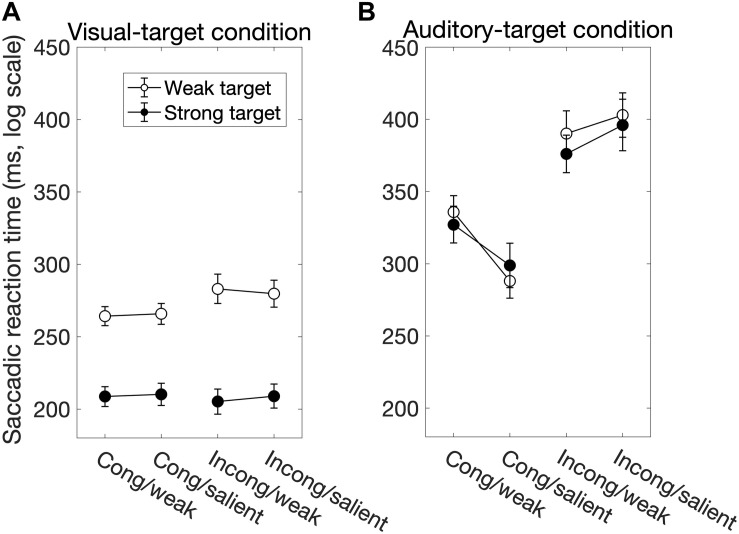
The factors influencing the saccadic reaction time (SRT) for the visual-target condition **(A)** and for the auditory-target condition **(B)**. White and black circles indicate the weak and strong targets, respectively. The error bars represent the standard error of the mean. For the statistical comparison of the factors, see Tables 1, 3. Generally, the saccade behavior depended on task modalities.

We found significant main effects of *Sal-target* [*F*(1,13) = 165.27, *p* < 0.0001], *Cong* [*F*(1,13) = 30.63, *p* = 0.0001] on SRT (Figure 2A). The main effect of *Sal-target* reflects the fact that the reaction time of a saccade toward the visual target significantly decreased as the target luminance increased. This is consistent with the previous studies (Bell et al., 2004; Marino et al., 2015). The effect of congruency indicates that the saccade toward a visual target is influenced by the spatial location of the auditory stimulus: the SRT was shorter when the positions of the auditory stimulus and the visual target were the same than when they were opposite to each other. The pupil effect suggests that the SRT is shorter for a large pupil size than for a small pupil size (Jainta et al., 2011; Wang et al., 2017). There was no significant effect of *Sal-nontarget*, indicating that the sound intensity had no impact on the saccade behavior in the visual-target condition.

**TABLE 3:** ANOVA results on SRTs for auditory task.

***Effect***	***F***	***p***	**$\eta_{\text{p}}^{2}$**
Sal-target	0.68	4.27	0.049
Cong	**129.70**	**<0.0001**	**0.91**
Sal-nontarget	**40.42**	**<0.0001**	**0.76**
Sal-target × Cong	0.34	0.57	0.026
Sal-target × Sal-nontarget	2.69	0.12	0.17
Cong × Sal-nontarget	**7.24**	**0.019**	**0.36**
Sal-target × Cong × Sal-nontarget	4.37	0.057	0.25

*The bold values mean statistical significance (p < 0.05).*

Interestingly, there was a significant interaction between *Sal-target* and *Cong* [*F*(1,13) = 6.70, *p* = 0.023]. A *post hoc* test showed that the effect of spatial congruency was significant only when the saliency of the target was weak; the RT was longer for incongruent non-target (*p* = 0.0002, Figure 4A).

**FIGURE 3:**
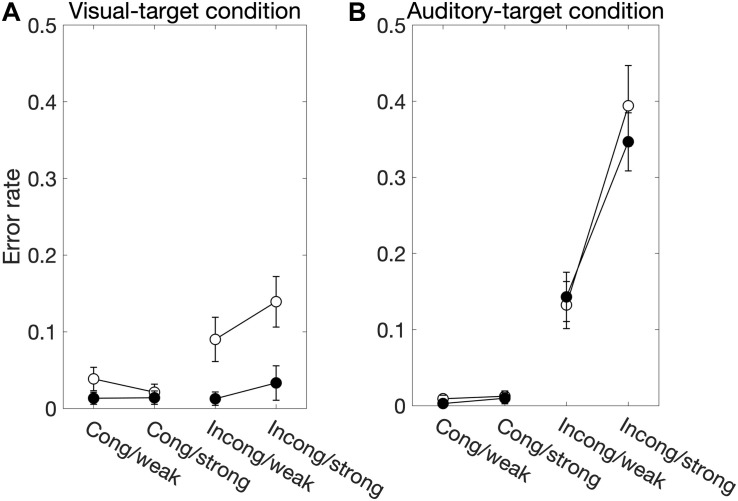
The factors influencing the error rate for the visual-target condition **(A)** and the auditory-target condition **(B)**. White and black circles indicate the weak and strong targets, respectively. For the statistical comparison of the factors, see Tables 2, 4. Generally, these results show effects similar to those in the SRT results.

We found significant main effects of *Sal-target* [*F*(1,13) = 8.17, *p* = 0.014], *Cong* [*F*(1,13) = 15.51, *p* = 0.0017] on error rates (Figure 3A). Error rates were calculated by dividing the number of error trials by the total number of valid trials. We also found a significant interaction between *Sal-target* and *Cong* [*F*(1,13) = 13.86, *p* = 0.0026], indicating that the distraction effect due to the incongruent auditory stimulus was larger for the weak target than for the strong one.

**TABLE 4:** ANOVA results on error rates for auditory task.

***Effect***	***F***	***p***	**$\eta_{\text{p}}^{2}$**
Sal-target	1.01	0.33	0.072
Cong	**55.38**	**<0.0001**	**0.81**
Sal-nontarget	**57.27**	**<0.0001**	**0.82**
Sal-target × Cong	0.41	0.53	0.03
Sal-target × Sal-nontarget	1.33	0.27	0.093
Cong × Sal-nontarget	**65.43**	**<0.0001**	**0.83**
Sal-target × Cong × Sal-nontarget	1.23	0.29	0.09

*The bold values mean statistical significance (p < 0.05).*

### Auditory-Target Condition

**FIGURE 4:**
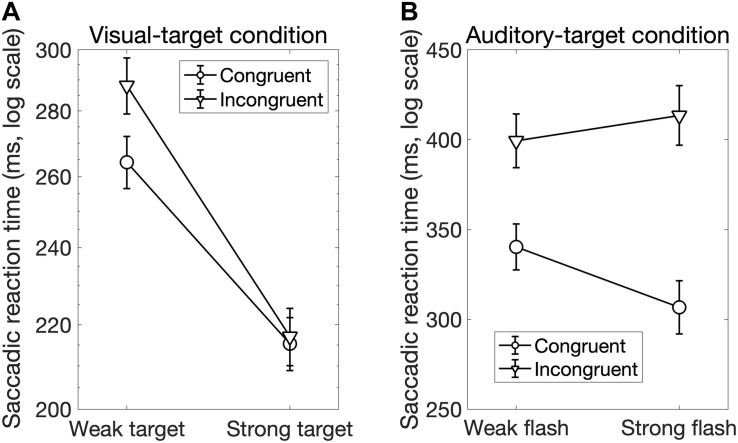
**(A)** Interaction between the target saliency and audiovisual spatial congruency in the visual-target condition. The factor of the non-target saliency was averaged in this figure. There was a spatial congruency effect on the SRT when the target luminance was low, whereas there was no spatial congruency effect when the target luminance was high. **(B)** Interaction between the audiovisual spatial congruency and non-target saliency (luminance of the visual stimulus) in the auditory-target condition. The factor of target saliency was averaged in this figure. The result shows that the enhancement effect caused by the congruent visual stimulus was greater (black dots in this figure and thick arrow in Figure 2) compared to the distraction effect caused by the incongruent visual stimulus (white dots in this figure).

We found significant main effects of *Cong* [*F*(1,13) = 129.69, *p* < 0.0001] and *Sal-nontarget* [*F*(1,13) = 40.42, *p* < 0.0001]. The main effect of congruency indicates that the congruent visual stimulus facilitated the SRT. The effect of *Sal-nontarget* indicates that the luminance of the visual stimulus was highly effective on the SRT even when the participants had to make a saccade toward the auditory target. Consistent with the insignificant effect of *Sal-nontarget* in the visual-target condition, we found no significant effect of *Sal-target* [*F*(1,13) = 0.67, *p* = 0.43]. This suggests that the intensity of the auditory target had no impact on SRT in the range of sound intensities tested in the present study. We found significant interactions between *Cong* and *Sal-nontarget* [*F*(1,13) = 7.24, *p* = 0.019]. This interaction indicates that the enhancement effect for the congruent condition (thick arrow in Figure 2B) was greater than the distraction effect for the incongruent condition (thin dashed arrow in Figure 2B): a *post hoc* analysis showed that there was a significant effect of visual saliency for the congruent condition [*F*(1,13) = 40.58, *p* < 0.0001, Figure 4B] but not for the incongruent condition [*F*(1,13) = 2.27, *p* = 0.16].

We found significant main effects of *Cong* [*F*(1,13) = 55.38, *p* < 0.001] and *Sal-nontarget* [*F*(1,13) = 57.27, *p* < 0.0001] on error rates (Figure 3B). We also found a significant interaction between *Cong* and *Sal-nontarget* [*F*(1,13) = 65.43, *p* < 0.0001], indicating that the distraction effect was larger for the strong visual stimulus than for the weak visual stimulus. The insensitivity of the SRTs to the visual saliency for the incongruent condition shown above might be caused by the smaller number of trials compared to the congruent condition.

In summary, first, the luminance of the visual stimulus had a large effect on SRT even for the auditory-target condition in which participants were forced to ignore the visual stimulus. Second, the influence of the auditory stimulus on SRT in the visual-target condition was more significant for the low-luminance visual target than for the high-luminance one. Third, no main effect of saliency of the auditory target was found. Fourth, the enhancement caused by the congruent visual stimulus had a larger effect than the distraction caused by the incongruent visual stimulus in the auditory-target condition. This enhancement was larger for the weak auditory target than for the strong auditory target.

### Pre-saccade Pupil Size

**FIGURE 5:**
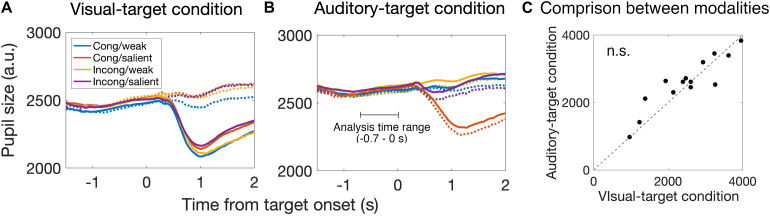
Time course of pupil size change around the target onset for the visual-target condition **(A)** and the auditory-target condition **(B)**. Solid and dashed lines indicate the strong and weak targets, respectively. Each color indicates the combination of the spatial congruency and the saliency of non-target stimulus. We analyzed the pupil data from –0.7 to 0 s relative to the target onset to examine the saccade preparation effect on pupil size. **(C)** Comparison of pre-saccade absolute pupil size between visual and auditory tasks. We found no significant difference between them, suggesting that there is no effect of task demand (i.e., engagement in the visual task vs. auditory task) on mean pre-saccade absolute pupil size. Each dot indicates data for each participant.

We analyzed the pre-saccade pupil size changes to examine how arousal states affect the saccade behavior and how they differed between the visual- and auditory-target conditions. Figure 5 shows the time course of the pupil size and the pupil derivative before and after target presentation. The constriction of the pupil size after target presentation is considered to be caused by pupillary light reflex and the apparent distortion in the camera image. The constriction was larger for the trials with strong visual stimuli than for those with weak visual stimuli. In the subsequent analysis, we focused only on the pre-saccade time range to examine the preparation of saccades.

**FIGURE 6:**
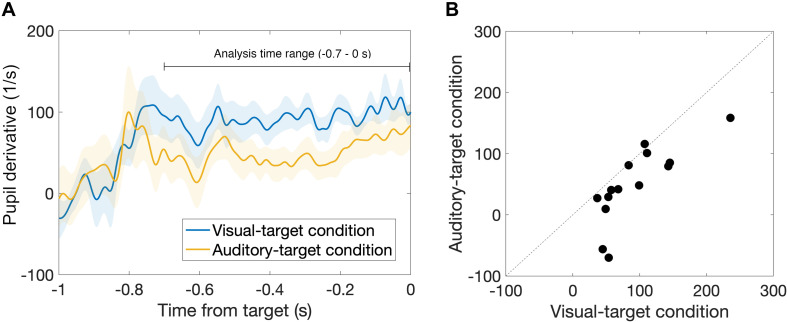
**(A)** Comparison of time courses of pre-saccade pupil derivative between visual- and auditory-target conditions. **(B)** Comparison of pre-saccade pupil derivative averaged within −0.7 to 0 s from target onset between visual- and auditory-target conditions. We found a significant difference between them, suggesting that, in contrast to absolute pupil size, pupil derivative is sensitive to the task set.

**FIGURE 7:**
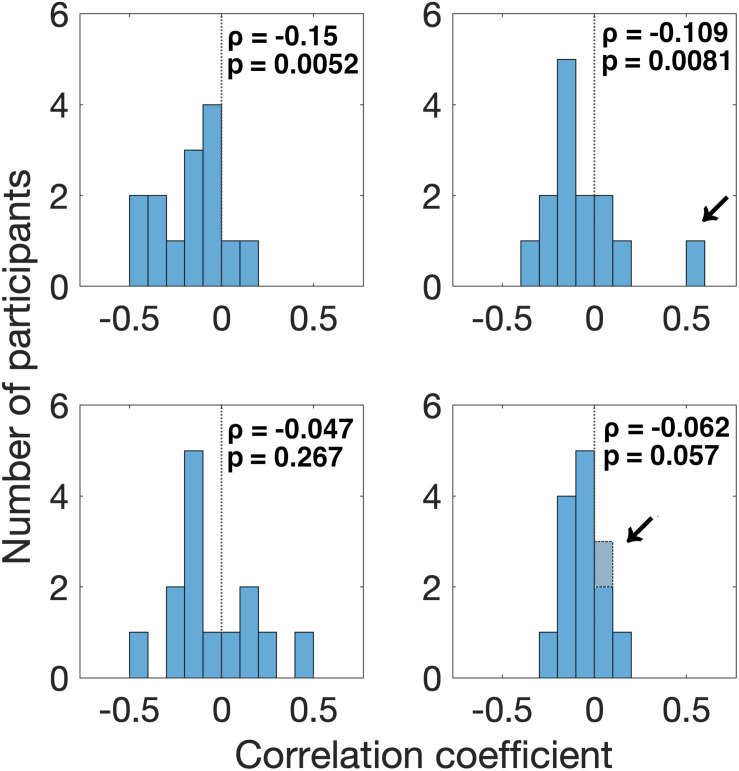
Distribution of correlation coefficients of SRT with pre-saccade absolute pupil size and derivative for all participants. We found that a significant proportion of participants (12/14) exhibited correlation coefficients smaller than zero for the relationship between SRT and absolute pupil size, in the visual-target condition. We also found that a significant proportion of participants (11/13) exhibited correlation coefficients smaller than zero for the relationship between SRT and pupil derivative in the visual-target condition. Note that we excluded the data from one outlier participant shown by an arrow for the analysis of pupil derivative. For the auditory-target condition, the effect was marginally significant for the relationship between SRT and pupil derivative.

First, we found that the pupil derivative during the pre-saccade time range was larger than zero for both visual- and auditory-target conditions (Figure 6). We also found a difference in the pupil derivative, but not in the absolute pupil size, between visual- and auditory-target conditions (Figures 5C, 6B). Second, we calculated Spearman’s rank correlation of the SRTs with the absolute pupil size and pupil derivative for each participant and for each target modality. We examined whether Spearman’s rank correlation coefficients (ρ) were larger than zero by applying Wilcoxon signed-rank test for Fisher’s z-transformed correlation coefficient. We found that a significant proportion of participants exhibited correlation coefficients smaller than zero between the absolute pupil size and SRTs only for the visual-target condition (mean ρ = −0.15,*p* = 0.0052, Figure 7).

**FIGURE 8:**
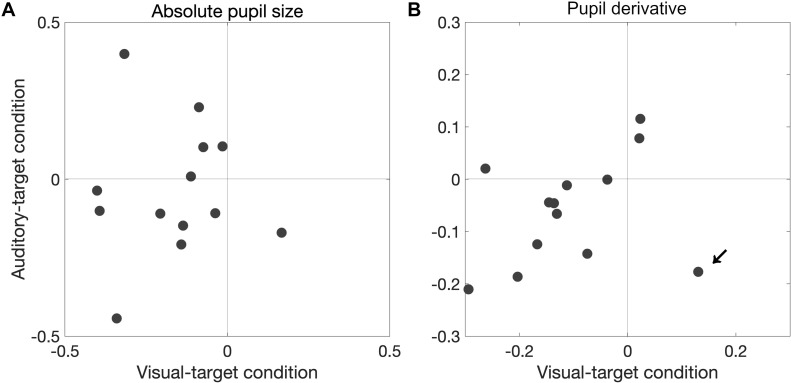
Consistency in pupil-SRT correlation between the visual- and auditory-target conditions for absolute pupil size **(A)** and for pupil derivative **(B)**. The correlations (of the correlation coefficients for the visual- and auditory-target conditions) were not significant for either absolute pupil size or the pupil derivative. However, when an apparent outlier participant (marked by the arrow; different from the one marked in Figure 7) was excluded, the correlation would became significant (Pearson’s *r* = 0.62, *p* = 0.037).

We also found that a significant proportion of participants showed correlation coefficients smaller than zero for the pupil derivative for the visual-target condition (mean ρ = −0.109,*p* = 0.0081). In this analysis, we excluded the data from one outlier participant which exceeded the 75th percentile +1.5× IQR (Interquartile range) for the visual-target condition (marked by an arrow in Figure 7). For the auditory-target condition, the effect was marginally significant (mean ρ = −0.062,*p* = 0.057), suggesting a possibility that the correlation between the pupil derivative and SRTs reflects a more general, supramodal effect of pupil-linked arousal on SRT. To examine the possibility more directly, we also examined whether the correlation between the pupil derivative and SRT was consistent for the visual- and auditory-target conditions within individual participants. The correlations (of the correlation coefficients for the visual- and auditory-target conditions) were not significant either for absolute pupil size of the pupil derivative (Figure 8A). Note that the scatter plot for the pupil derivative shows a trend of a linear relationship: When an apparent outlier participant (marked by the arrow in Figure 8B; different from the one marked in Figure 7) was excluded, the correlation became significant correlation (Pearson′s *r* = 0.62,*p* = 0.037).

Finally, we examined the possibility of an “inverted-U-like” effect, which is a nonmonotonic relationship between pupil size and behavioral performance suggested in Aston-Jones and Cohen (2005). For that purpose, we attempted to fit a second-order polynomial function to the relationship between pupil and SRT data. The result failed to support the possibility: the 2nd order coefficients did not significantly differ from zero (*ps* > 0.20),and the coefficients of determination were low for all conditions and for both the absolute pupil size (*R*^2^*s* < 0.10)and pupil derivative (*R*^2^*s*≤0.26).

## Discussion

### Saccade Behavior Differed Between Visual and Auditory Modalities

The present study demonstrated that the characteristics of SRT were different between the visual- and auditory-target conditions in the mean SRT, the effect of stimulus saliency, and the effect of pupil size. The results imply that we should not naively treat the saccadic movements to the auditory target as an indicator of auditory spatial attention in the same way as in the visual modality.

The SRT for the visual-target condition was generally shorter than that for the auditory-target condition. This may be due to the difference in the distance between the internal representations of the fixation and target locations in the two modalities. It should be noted, however, that earlier studies (Frens and Van Opstal, 1995; Gabriel et al., 2010) showed that the SRTs in the case of a ten-degree target position were comparable for the visual and auditory targets. In addition, auditory-targeting saccades have shorter SRTs in more natural conditions, compared to visual-targeting saccades (Corneil et al., 2002). It was also possible that the subjective saliency of the visual stimulus was larger than that of the auditory stimulus. The perceptual dominance of the visual stimulus might obscure the relatively subtle effect of the auditory target/non-target presented simultaneously. Thus, interpretation needs to be careful, as some other factors such as the stimulus contrast level difference between modalities could influence their results (e.g., the correlation between SRTs and pupil size). More studies are needed, in which the saliencies of the auditory and visual stimuli should be carefully equalized.

In the following sections, we discuss the results for the visual- and auditory-target conditions separately.

### Potential Physiological Mechanisms Underlying the Effect of Target Saliency and the Congruency of Target and Non-target

**FIGURE 9:**
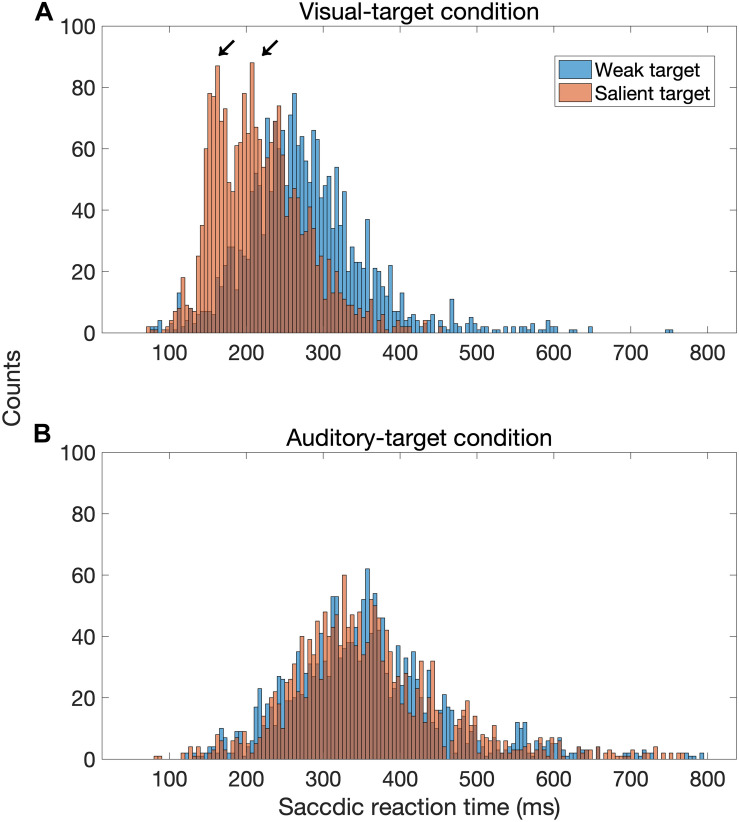
Distribution of saccadic reaction time (SRT) for the visual-target condition **(A)** and the auditory-target condition **(B)**. In this figure, factors other than target saliency were combined. The distribution seems to be bimodal when the visual target was strong (red bars) compared to when it was weak (blue bars), whereas there is no such a tendency for the auditory-target condition.

Our finding that the visual stimulus intensity modulates the saccadic behavior is consistent with the previous studies (Bell et al., 2006; Marino et al., 2015). Marino et al. (2015) showed that the target luminance altered the visual responses in the superficial SC and the amount of buildup activity in the dSC. These modulations influence the SRT and the likelihood of express saccade. The characteristics of the dSC neurons may also be able to account for the lack of target-intensity effect in the auditory-target condition. Neurons in the dSC may be less sensitive to the sound intensity, and the threshold intensity is more variable compared with the other nuclei in the auditory processing pathway (Hirsch et al., 1985). It was interesting that in the visual-target condition, the congruency of the auditory non-target was observed only when the visual target had a lower luminance. This may be accounted for by assuming two pathways control saccade latency. Previous studies (Bell et al., 2006; Marino et al., 2015) suggested that high-intensity visual stimuli evoke express saccades, which have been traditionally defined by the presence of a bimodal distribution in the SRT histogram (Fischer and Weber, 1993). The express saccade in humans is considered to be 100–120 ms (Fischer and Ramsperger, 1984). The present result can be accounted for by making two assumptions: (1) the relative contribution of express-like saccade increases as target saliency increases and (2) the pathway of the express saccade is not influenced by auditory sensory information. In fact, the SRT histogram shows a bimodal distribution for the strong visual target, but not for the weak visual target (Figure 9). We statistically tested the number of modes in the distribution of SRTs with the Silverman test (Silverman, 1981). We used the R package for this test (Schwaiger and Holzmann, 2013). This method tests the null hypothesis that a density distribution has at most k modes. For example, if the *p*-value is lower than 0.05 for a tested hypothesis of *k* = 2, its distribution is considered to have three or more modes. We found that the distribution for the strong visual target had two modes (the null hypothesis for one mode was rejected: *p* = 0.0054, but not rejected for two modes: *p* = 0.53), whereas none of the null hypotheses for the distributions in the other conditions were rejected (for the weak visual target, *p* = 0.10 for one mode; for the weak auditory target, *p* = 0.096 for one mode; for the strong auditory target, *p* = 0.056 for one mode).

A neural pathway specialized for express saccade was proposed by Isa (2002). In this model, if the visual response in the superficial SC (sSC) is large enough and the gap between fixation disappearance and target onset is long enough to evoke buildup activity reaching a certain value, the information of the visual input directly projects to the dSC from the sSC without passing through the frontal eye field (FEF) or higher cortical pathways where auditory cortical processes might contribute. It is possible that that the pathway with a direct projection from sSC to dSC is not influenced by auditory signals.

### Link Between Pupil Size and Saccade Behavior

We found that the pre-saccade pupil derivative reflects the effect of task set (visual vs. auditory tasks). Recent studies have shown that the measure of pupil derivative, rather than the absolute size, reflects more specifically the activity of the locus coeruleus (LC) (Reimer et al., 2014, 2016; McGinley et al., 2015). Reimer et al. (2016) showed that the pupil derivative correlates with cortical norepinephrine (NE) activity. This leads us to infer that the visual-target condition, which exhibited the greater pupil derivative in the present result, was accompanied by higher NE activity during the task. This notion that the arousal level was higher for the visual-target condition than for the auditory one is consistent with the fact that the mean SRT was shorter for the visual-target condition. On the other hand, we found no significant difference in the pre-saccade absolute pupil size between visual- and auditory-target conditions. These results suggest that the absolute pupil size is less sensitive to modality-specific arousal effects.

Interestingly, we found the correlation between the absolute pupil size (not the pupil derivative) and SRTs only for the visual-target condition. Why was there a correlation only for the absolute pupil size in the present study? According to the neurophysiological study by Reimer et al. (2016), the absolute pupil size and pupil derivative may reflect different neural processes. Long-lasting pupil dilation (corresponding to the absolute pupil size in the present study) is accompanied by sustained activity in cholinergic axons. They also found that the cholinergic activity matched the period of movement, while NE activity matched the moment-to-moment fluctuations in pupil dilation, suggesting that the premotor or motor-related activity correlates with the absolute pupil size. Several studies have reported that the injection of cholinergic agonist nicotine into the SC increases the frequency of express saccade (Aizawa et al., 1999; Watanabe et al., 2005). Possibly, the correlation between the absolute pupil size and SRT can be explained if we assume an association between the cholinergic activity and task modalities, because there is a link between pupil size and the SC (Wang et al., 2012, 2014; 2015). This interpretation is in line with the present result showing bimodal peaks in the visually evoked saccades but not in the auditory evoked saccades (Figure 9). Note also a study indicating that the SC shows a lesser increase in pre-target activity for an auditory target than for a visual target (Bell et al., 2004; see “Introduction” section). It is not likely that the pathway connecting pupil-size modulation and pre-stimulus activity in the SC was activated by the stimulus *per se*, since the visual and auditory stimuli were presented at the same time, and thus the sensory input was essentially the same during the visual and auditory tasks. Rather, the pathway may be activated by the participant’s directing attention toward visual objects. The present result showing the effect of task set on pupil derivative (Figure 6) is consistent with this idea. On the other hand, the consistency between visual and auditory modalities for the pupil derivative and SRT link suggests a general supramodal effect of pupil-linked arousal or preparatory activity on SRT. In summary, the pupil derivative reflects the task set and a general arousal effect, while the absolute pupil size reflects visual-modality-specific activity. Future studies are needed to clarify how the absolute pupil size and pupil derivative differently reflect the neural activities.

Another possible explanation is that the discrepancy in the effects of pupil size on SRTs between task modalities is due to non-cognitive factors that differed between task modalities. Compared to pupil derivative, the measure of absolute pupil size may potentially be affected by non-cognitive factors such as drifts in participant head position or gradual habituation to ambient light. The difference in the amount of contributions of such non-cognitive factors could obscure a correlation in the auditory-target condition that might exist.

## Data Availability Statement

The original contributions presented in the study are included in the article/supplementary material, further inquiries can be directed to the corresponding author.

## Ethics Statement

The studies involving human participants were reviewed and approved by the Research Ethics Committee of Nippon Telegraph and Telephone (NTT) Communication Science Laboratories. The patients/participants provided their written informed consent to participate in this study.

## Author Contributions

SY and SF designed the research and wrote the article. SY performed the experiments and analyzed the data. Both authors contributed to the article and approved the submitted version.

## Conflict of Interest

Both authors are employees of NTT Communication Science Laboratories, which is a basic-science research section of Nippon Telegraph and Telecommunication corporation (NTT). This does not alter the authors’ adherence to policies of Frontiers.
